# Follow our path with asparaginase activity: one technique, but different uses in clinical practice

**DOI:** 10.1186/s40164-022-00351-5

**Published:** 2022-11-04

**Authors:** Daiane Keller Cecconello, Ciliana Rechenmacher, Klerize Anecely de Souza Silva, Fernanda Fetter Scherer, Thomas Dal Bem Prates, Rebeca Ferreira Marques, Liane Esteves Daudt, Mariana Bohns Michalowski

**Affiliations:** 1 Post Graduate Program in Child and Adolescent Health, Universidade Federal do Rio Grande do Sul, Porto Alegre, RS Brazil; 2 Translational Pediatrics Laboratory, Experimental Research Center, Hospital de Clínicas de Porto Alegre, Porto Alegre, RS Brazil; 3 Hospital de Clínicas de Porto Alegre, Porto Alegre, RS Brazil

**Keywords:** Acute lymphoblastic leukemia, PEG asparaginase, Enzimatic activity, Desensitization protocols

## Abstract

Acute lymphoblastic leukemia is the most common childhood malignancy. One of the drugs used in the treatment is Asparaginase, and monitoring of its activity levels enables better outcomes. Since 2018, our laboratory has been working to establish a regular analysis of activity. This implementation allowed to qualify care by detecting silent inactivation and also establishing desensitization as a safe way to overcome the lack of *Erwinia*. We were able to monitor children aged 0 to 18 years who were being treated with PEG-ASNase. The activity was assessed on days 7 (90 samples) and 14 (52 samples) after ASNase infusions. 142 samples were analyzed. 95.7% reached an adequate activity level (≥ 0.1 IU/mL). Patients treated with ASNase can develop allergic reactions. With the activity monitoring, is possible to circumvent situations like these and implement desensitization protocols for patients who had clinical hypersensitivity without inactivation. Desensitization induces temporary unresponsiveness to drug antigens, allowing the patients to proceed with the prescribed chemotherapy. We have received samples from four patients being treated with different desensitization protocols. Patients tolerated the protocols well. Only one had a grade 2 reaction during the infusion and activity < 0.1 IU/mL, which resulted in the switch to *Erwinia*. The dose adaptation is a possible and more recent use of ASNase monitoring and we were able to confirm the feasibility of PEG-ASNase desensitization protocols.

## To the Editor,

Acute lymphoblastic leukemia (ALL) is the disease most commonly seen in children [1]. Due to current chemotherapy regimens, long-term results have improved, being associated with event-free survival and overall survival rates around 80% and close to 90%, respectively [2]. One of the drugs used in the treatment is Asparaginase (ASNase), and the monitoring of its activity levels has allowed for better outcomes [1].

Since 2018, our laboratory has been working to establish a regular analysis of ASNase activity in children being treated in Brazil. Moreover, the data in this study demonstrated that this implementation supported care improvement by detecting silent inactivation [3, 4].

**Fig. 1:**
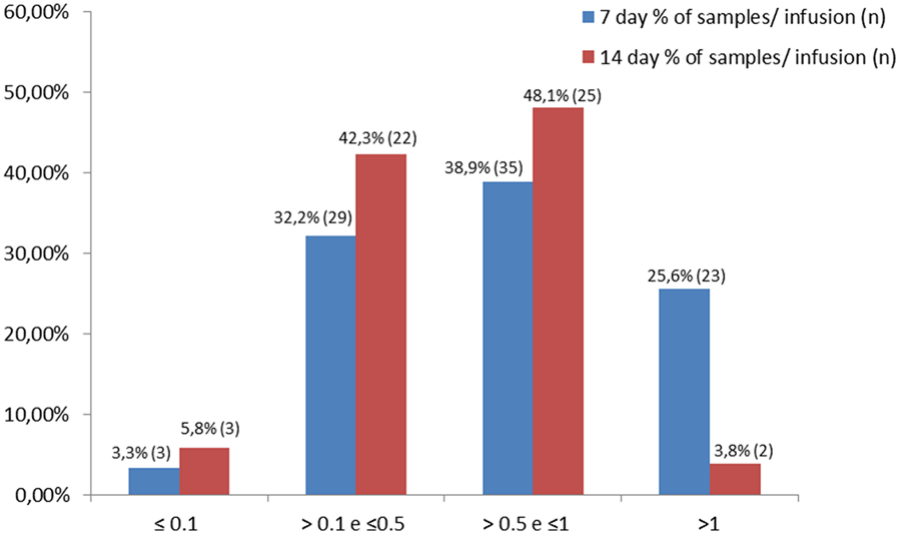
Enzymatic activity (IU/mL) distribution assessed 7 and 14 days after infusion (n = 142 samples). Medians (IQR) were 0.654 (0.420–1.017) and 0.512 (0.321–0.796) on days 7 and 14, respectively. Values were distributed according to the % of samples and the number of infusions on days 7 and 14. IQR: Interquartile range

We were able to monitor children aged 0 to 18 years who were being treated for ALL with PEG-ASNase. The activity was assessed after infusions on days 7 (90 samples) and 14 (52 samples) during the first and second infusions of the BFM 2009 protocol induction. As shown in Fig. 1, 142 samples were analyzed, out of which 95.7% (136) reached an adequate activity level (≥ 0.1 IU/mL) and only 4.3% (6) had levels lower than expected. These data agree with those described in the literature [5].

Patients treated with ASNase may develop allergic reactions [4]. With activity monitoring, it is possible to avoid situations like this and to implement desensitization protocols for patients who had clinical hypersensitivity to PEG-ASNase without inactivation.

We received samples suspected of having allergy/inactivation from other centers in Brazil. These were monitored with the use of desensitization protocols. These induce temporary unresponsiveness to drug antigens, allowing the patients to proceed with the chemotherapy to which they had a reaction [6]. Desensitization is useful where *Erwinia* is not easily available.

We have received samples from four patients being treated with different desensitization protocols. The characteristics are in Table 1. There is still limited knowledge on PEG-ASNase desensitization. Concha et al. reported a successful protocol used in five patients. They suggest that patients might benefit from this viable alternative to drug discontinuation [7]. Their protocol is similar to described by Verma et al. [8].

**Table 1:** Characteristics of patients undergoing desensitization protocols

Patient	1	2	3	4
Gender	Male	Male	Female	Female
Age (years	9	5	11	13
Diagnosis	B-ALL	B-ALL	T-ALL	T-ALL
Treatment protocol	UK ALL R3	IC-BFM 2009	IC-BFM 2009	IC-BFM 2009
Previous allergy	Yes	Yes	Yes	Yes
CTCAE grade	4	2	2	2
Desensitization protocol	Premedication: cetirizine, famotidine, montelukast, and methylprednisolone. Bags of saline with progressively increasing doses and infusion rates	PEG 2500 UI/m^2^ diluted in 1000 mL of saline. Premedication: hydrocortisone, promethazine, montelukast, and cetirizine	Premedication: promethazine and hydrocortisone	H2 and H1 blockers and corticosteroid pretreatment. 3 bags of saline with different dilution rates: 1:1, 1:10, 1:100
Symptoms during desensitization	No	Yes	No	No
D7 activity levels (IU/mL)	0.43	0.08	0.72	0.91
D14 aActivity levels (IU/mL	NA	0.03	0.64	0.34
Symptoms improved	Yes	Yes	Yes	Yes

*ALL* Acute lymphoblastic leukemia, *UK* United Kingdom, *BFM* Berlin-Frankfurt-Münster group, *IC* Intercontinental, *CTCAE* Common Terminology Criteria for Adverse Events, *NA* Not available

Patients who had allergic reactions may choose to undergo a rechallenge protocol with premedication, switch to *Erwinia*, or discontinue the therapy. In this study, patients tolerated the protocols. Only one (#2) had a grade 2 reaction and activity < 0.1 IU/mL during the infusion, which resulted in the switch to *Erwinia*.

Similar to described by Verma et al., PEG-ASNase can be administered to patients who had hypersensitivity using desensitization protocols. Most patients sustained levels of activity, making it a cost-effective option [8], As reported by Swanson et al., in patients who presented angioedema, vomiting, and positive antibodies in the infusion process before undergoing the desensitization protocol, this failed more. Therefore, attention should be paid to this group [9].

The issue is deciding whether to use a desensitization protocol or switch to *Erwinia*. The protocol in patients with hypersensitivity should be applied with regular monitoring, as this helps to prevent subtherapeutic activity from occurring. Tong et al. showed that patients with inactivation who continued the treatment with PEG-ASNase had a decrease in antibodies and started to show therapeutic activity later. As the recovery of ASNase activity may take an unpredictable amount of time, we recommend switching to *Erwinia* instead of using desensitization approaches. Patients with PEG-ASNase inactivation should continue taking this drug only if *Erwinia* is not available [10].

Dose adaptation is a recent use of ASNase monitoring. As described by Tong et al., patients who were not allergic to PEG-ASNase had a mean activity level of 0.899 IU/mL. They observed that if patients did not have allergy or inactivation, the use of a regimen of 2500 IU/m^2^ led to high serum levels [11]. They reported that dose reduction may be possible, as they used protocols that reduced the dose of PEG-ASNase to 1000 IU/m^2^, and approximately 80% of patients had adequate activity (> 0.1 IU/mL) [12].

We were able to demonstrate how a simple technique can be efficiently incorporated into the treatment of ALL, improving the care of patients. Our data on silent inactivation correlated with those described in the literature. We were able to confirm the feasibility of desensitization protocols in patients who had clinical allergy but no drug inactivation. The impact of dose adjustments on possible adverse effects remains to be studied.

## Data Availability

Yes.
